# Tuning the Electronic and Optical Properties of the Novel Monolayer Noble-Transition-Metal Dichalcogenides Semiconductor β-AuSe via Strain: A Computational Investigation

**DOI:** 10.3390/nano12081272

**Published:** 2022-04-08

**Authors:** Qing-Yuan Chen, Bo-Run Zhao, Yi-Fen Zhao, Hai Yang, Kai Xiong, Yao He

**Affiliations:** 1 School of Physical Science and Technology, Kunming University, Kunming 650214, China; borunzhao@163.com (B.-R.Z.); zyfen0402315@163.com (Y.-F.Z.); kmyangh@263.net (H.Y.); 2 Materials Genome Institute, School of Materials and Energy, Yunnan University, Kunming 650091, China; xiongkai@ynu.edu.cn; 3 Department of Physics, Yunnan University, No. 2 Green Lake North Road, Wu Hua Qu, Kunming 650091, China

**Keywords:** two-dimensional materials, β-AuSe, structural property, electronic property, optical property, strain effect, *p–d* hybridization effect

## Abstract

The strain-controlled structural, electronic, and optical characteristics of monolayer β-AuSe are systematically studied using first-principles calculations in this paper. For the strain-free monolayer β-AuSe, the structure is dynamically stable and maintains good stability at room temperature. It belongs to the indirect band gap semiconductor, and its valence band maximum (VBM) and conduction band minimum (CBM) consist of hybrid Au-*d* and Se-*p* electrons. Au–Se is a partial ionic bond and a partial polarized covalent bond. Meanwhile, lone-pair electrons exist around Se and are located between different layers. Moreover, its optical properties are anisotropic. As for the strained monolayer β-AuSe, it is susceptible to deformation by uniaxial tensile strain. It remains the semiconductor when applying different strains within an extensive range; however, only the biaxial compressive strain is beyond −12%, leading to a semiconductor–semimetal transition. Furthermore, it can maintain relatively stable optical properties under a high strain rate, whereas the change in optical properties is unpredictable when applying different strains. Finally, we suggest that the excellent carrier transport properties of the strain-free monolayer β-AuSe and the stable electronic properties of the strained monolayer β-AuSe originate from the *p–d* hybridization effect. Therefore, we predict that monolayer β-AuSe is a promising flexible semiconductive photoelectric material in the high-efficiency nano-electronic and nano-optoelectronic fields.

## 1. Introduction

With the development of investigations into new energy and photoelectric fields, researchers’ demand for the high performance of materials is increasing [1,2,3,4,5,6]. The research into and application of two-dimensional materials, a promising new field, have seen significant progress in recent years. More and more two-dimensional (2D) materials have been designed and prepared. According to the individual characteristics of different 2D materials, they can be divided into different types. Because of their distinct functional characteristics, 2D materials have attracted much attention in the new energy and photoelectric fields [7,8,9,10,11,12,13,14,15,16,17,18,19,20,21]. Among them, 2D transition-metal dichalcogenides (TMDs) are essential. Due to the diversity of its constituent elements, the family members of TMDs are numerous [9,10,11,12]. There are some outstanding characteristics of TMDs in common, such as band gaps that cover the visible and near-infrared light (1.1–2.5 eV) regions, strong Coulomb interactions, and strong light-matter coupling, which leads to its wide application in new energy and photoelectric fields, such as solar cells, photocatalysis, luminescent materials, and photodetectors [9,10,11,12,13,14]. However, low carrier mobility (about 200–500 cm^2^ V^−1^ s^−1^) is one of the factors limiting the development of TMDs. Therefore, finding and designing novel TMDs with excellent photoelectric properties and high carrier mobility has become an important research direction. To achieve this purpose, researchers mainly adopt two methods. One is searching for and designing new 2D materials with excellent photoelectric properties and high carrier mobility in other types of 2D materials besides TMDs. The other is continuously exploring novel TMD materials or modifying existing materials to meet expectations.

In recent years, a new 2D material, named noble-transition-metal chalcogenides (NTMDs), composed of noble-transition-metal elements and chalcogenides, has been discovered. Up to now, scientists have theoretically predicted or experimentally prepared NTMDs such as PtTe_2_, PdTe_2_, PtSe_2_, PtS_2_, PdS_2_, and PdSe_2_ [22,23,24,25,26,27,28]. These new 2D NTMDs are rich in *d*-electrons and exhibit some special properties such as strong interlayer interaction, unique pentagonal structure, diverse phases, high mobility and stability, and largely tunable electronic structures, which offset the deficiencies of traditional TMDs and black phosphene. Therefore, NTMDs have good application prospects in the field of new energy and optoelectronics [22,23,24,25,26,27,28,29]. However, because they are a new type of 2D material, there is scarce research on NTMDs. Hence, systematic research on NTMDs is important.

Recently, a novel 2D material consisting of noble-transition-metal Au and chalcogenide Se has been reported. In 2019, Gong et al., using first-principles calculations, predicted that the structure of 2D β-AuSe is stable, and its suitable band gap leads to its good response to the light in the visible region. In addition, compared with traditional TMD materials, 2D β-AuSe has an exceptionally high carrier mobility (about 10^3^–10^5^ cm^2^ (V s)^−1^). More importantly, the 2D β-AuSe exhibits in-plane anisotropy in light absorption coefficient, photoconductivity, and carrier mobility. All of the above attractive properties make few-layer β-AuSe a promising candidate for polarization-sensitive photodetectors, synaptic devices, microdigital inverters, channel materials in field-effect transistors, and solar cells [26]. In the same year, Machogo et al. adopted Gold(III) chloride hydrate as a precursor for prepared 2D β-AuSe using the colloidal synthesis method. They employed X-ray electron spectroscopy (XPS), Raman spectroscopy, transmission electron microscopy (TEM), and ultraviolet-visible (UV-VIS) spectroscopy to observe its structural properties. Furthermore, they found that the broad absorbance bands in the UV-VIS spectra of both α-AuSe and β-AuSe showed localized surface plasmon resonance [30]. In the same year, Bai et al. used the DFT method to study the structural properties and band structure of β-AuSe. The β-AuSe showed excellent dynamic and thermodynamic stability, and its formation energy reached −7.87 eV/atom [31]. In 2020, Tang et al. calculated the in-plane anisotropy of ultra-high carrier mobility for β-AuSe using ab initio calculation. They found that the electrons prefer to conduct along the square-planar (***a***) direction with mobility up to 3.98 × 10^4^ cm^2^ (V s)^−1^ and the hole mobility along the linear (***b***) direction reaches 8.03 × 10^3^ cm^2^ (V s)^−1^, which is about 40 times higher than that along the ***a***-direction, which reveals the contributions of the two nonequivalent Au channels on its electronic properties [32]. Its application in the field of high-efficiency optoelectronic devices was confirmed. In 2021, Yin et al. used the first-principles method to design the photocatalytic hydrogen evolution reaction’s Z-scheme heterostructures of AuSe/SnS. This kind of heterostructure can respond to the most visible and ultraviolet light, and its solar-to-hydrogen (STH) efficiency reaches 23.96% [33]. Therefore, as a new kind of 2D NTMD material, 2D β-AuSe has a bright future in the new energy and photoelectric field.

This paper investigates the strain-controlled structural and photoelectric properties of the monolayer β-AuSe using first-principles calculations. We find that monolayer β-AuSe is dynamically stable and keeps good stability at room temperature. As for electronic properties, strain-free monolayer β-AuSe belongs to the indirect band gap semiconductor. Its valence band maximum (VBM) and conduction band minimum (CBM) are hybrid Au-*d* and Se-*p* electrons. The band gap of monolayer β-AuSe is 1.21 eV and 1.8 eV using the Perdew-Burke-Ernzerhof (PBE) and Heyd-Scuseria-Ernzerhof (HSE06) methods, respectively. The Au–Se bond consists of a partial ionic bond and a partial polarized covalent bond. Meanwhile, lone-pair electrons exist around Se and lie between different layers. Moreover, the strained monolayer β-AuSe remains the semiconductor when applying different strains within an extensive range; however, only the biaxial compressive strain beyond −12% leads to a semiconductor–semimetal transition. As for the optical properties, strain-free monolayer β-AuSe are anisotropic. Furthermore, its optical absorption properties in the ultraviolet (UV) region are better than in the visible (VIS) region. However, the reflection properties differ in the UV and VIS regions and in different directions. In addition, changes in optical properties vary under different strains. Finally, we suggest that the strain-free monolayer β-AuSe’s excellent carrier transport properties and the strained monolayer β-AuSe’s stable electronic properties originate from the *p–d* hybridization effect. Hence, it is a promising flexible semiconductive photoelectric material in the field of polarization-sensitive optoelectronics, solar-blind photoelectric detection, environmental monitoring, high-efficiency directional optoelectronics, and even ultraviolet communication.

## 2. Materials and Methods

In this paper, we used the version 5.4.1 of Vienna ab initio simulation package (VASP) [34,35], which is based on the density functional theory (DFT), to accomplish all of the first-principles calculations. VASP is a software package created, distributed, and maintained by the Hafner Research Group at the University of Vienna in Vienna, Austria. The projector-augmented wave approximation method was adopted to manage the interaction between electrons [35,36]. The plane wave energy cutoff was chosen to be 600 eV. The generalized gradient approximation according to Perdew, Burke, and Ernzerhof (GGA-PBE) approximation method was chosen to deal with the exchange correlation energy [34,37]. In order to verify the accuracy of the calculated results, the HSE06 hybrid functional method [38,39] with high precision was employed to calculate the electronic and optical properties. Based on previous studies, we know that the spin-orbit coupling (SOC) effect only shrinks the calculated band gap slightly [26,31,32,33]. Therefore, in our study, the electronic and optical properties of monolayer β-AuSe were calculated without the SOC effect.

**Figure 1:**
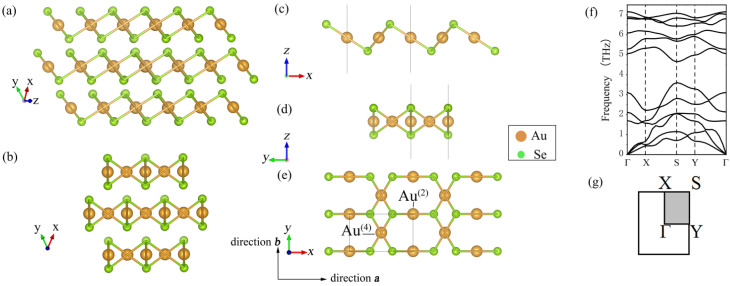
(**a**,**b**) The crystal structure of the bulk β-AuSe. (**c**–**e**) The crystal structure of the monolayer β-AuSe. The unit cell is in the black framework. (**f**) The phonon dispersion spectrums for the monolayer β-AuSe. (**g**) The Brillouin zone of the monolayer β-AuSe.

The 2D monolayer β-AuSe structure in our calculation was stripped from the bulk β-AuSe (Figure 1a,b). In order to eliminate the interaction between different layers of 2D β-AuSe in the ***z*** direction and better simulate the monolayer material, a vacuum layer of 30Å was added to our model in the ***z*** direction (Figure 1c–e). The structure in the black framework in Figure 1 corresponds to the primitive cell of monolayer β-AuSe. There were two types of Au atoms in the structure: Au atoms connected with four Se atoms were named Au^(4)^, and Au atoms connected with two Se atoms were called Au^(2)^.

We adopted Gamma-central K-points with a grid size of 16 × 27 × 1 in the Brillouin region. The energy convergence criterion adopted in our structural optimization calculation was 10^−7^ eV.

The bulk and monolayer β-AuSe in the calculation are shown in Figure 1a–e. The structural parameters are in accordance with earlier research [24,32,33].

We used both the PBE method and the HSE06 hybrid functional method to calculate the related optical properties. First, the imaginary part of the frequency-dependent dielectric function of the monolayer β-AuSe was calculated from Equation (1) [40]. In this equation, *ε*_2_ refers to the imaginary part of the frequency-dependent dielectric function; *c* and *v* refer to conduction and valence band states, respectively; and *u_ck_* is the cell periodic part of the wavefunctions at the *k*-point *k*.

(1) $$\varepsilon_{2}(\omega )=\frac{4\pi^{2}e^{2}}{\Omega }\lim_{q\to 0}\frac{1}{q^{2}}\sum_{c,v,k}2w_{k}\delta (\varepsilon_{ck}-\varepsilon_{vk}-\omega )\times \langle u_{ck+e_{\alpha }q}|u_{vk}\rangle {\langle u_{ck+e_{\beta }q}|u_{vk}\rangle }^{*}.$$

Next, the real part of the dielectric function (*ε*_1_) was calculated according to the Kramers–Kronig relation, as shown in Equation (2). Index *P* denotes the principal value [40].

(2) $$\varepsilon_{1}(\omega )=1+\frac{2}{\pi }P\int_{0}^{\infty }\frac{\varepsilon_{\alpha \beta }^{\left(2\right)}(\omega \prime )\omega \prime }{\omega \prime^{2}-\omega^{2}+i\eta }d\omega \prime$$

Then, we calculated its most important optical characteristics, such as the reflection coefficient and absorption coefficient, according to Equations (3)–(5) [41]. Here, *R(ω)* indicates the reflection coefficient, I*(ω)* indicates the absorption coefficient, *c* indicates the velocity of light, and *ε(ω)* indicates the frequency-dependent dielectric function.

(3) $$\varepsilon (\omega )=\varepsilon_{1}(\omega )+i\varepsilon_{2}(\omega ),$$

(4) $$R(\omega )=|\frac{\sqrt{\varepsilon (\omega )-1}}{\sqrt{\varepsilon (\omega )+1}}{|}^{2}=\frac{{\left(n-1\right)}^{2}+k^{2}}{{\left(n+1\right)}^{2}+k^{2}},$$

(5) I(ω)=( 2)ωc [ε1(ω)2+ε2(ω)2−ε1(ω)]12,

## 3. Results

### 3.1. Properties of Monolayer Strain-Free β-AuSe

#### 3.1.1. Structural Properties

First, we calculated the phonon spectrum of the bulk β-AuSe to verify the stability of the bulk β-AuSe (Figure S1). We found that the bulk β-AuSe is dynamically stable owing to the nonexistence of imaginary frequency in the entire BZ, which indicates that the monolayer β-AuSe can be obtained by stripping from the bulk materials.

Secondly, we found that there is no imaginary frequency in the whole Brillouin zone in the calculated phonon spectrum for monolayer β-AuSe (Figure 1f,g), which agrees well with previous results (Tang [32] and Gong [26]). Additionally, the ab initio molecular dynamics simulation (AIMD) is shown in Figure S2. The results confirm that the structure of monolayer β-AuSe established in our study is correct. Meanwhile, the results indicate that monolayer β-AuSe has good dynamic stability and stability at room temperature.

#### 3.1.2. Electronic Properties

Electronic properties such as the band structure, density of states, and charge density are the main characteristics studied in 2D materials. In this study, we investigated the electronic properties of monolayer β-AuSe in depth. This study could help us explore the excellent electronic properties of 2D β-AuSe and explain the mechanisms of its outstanding optical properties.

**Figure 2:**
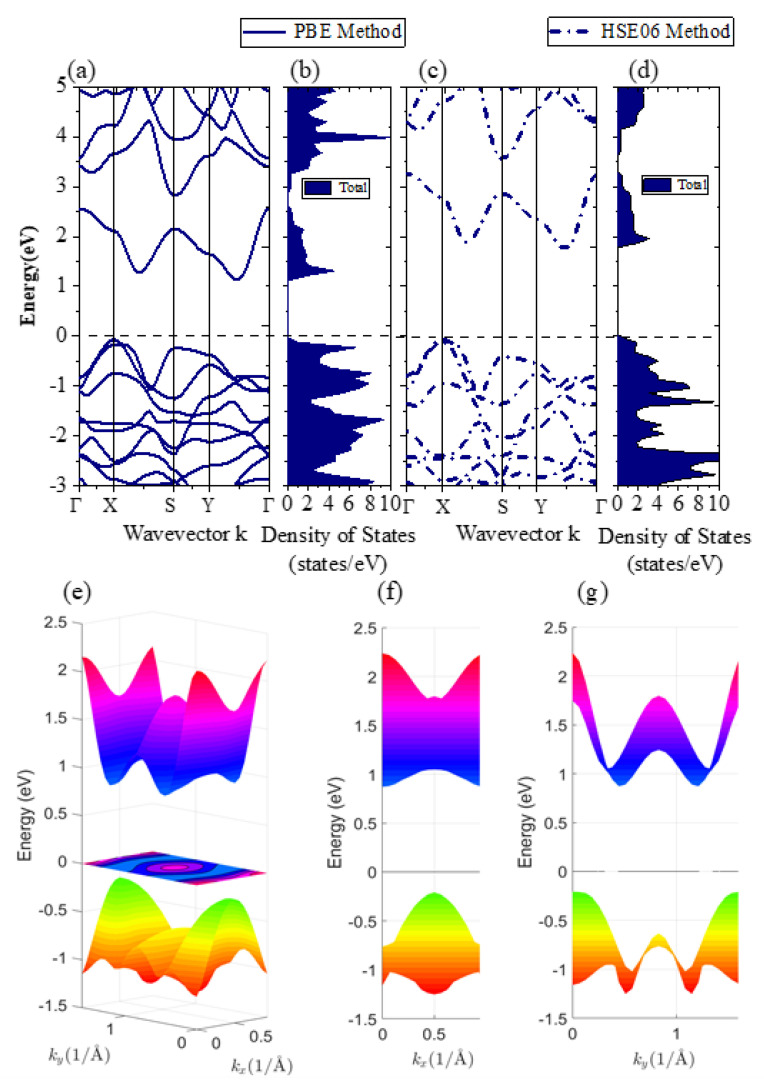
(**a**,**c**) The band structure of the monolayer β-AuSe using the PBE method and HSE06 method, respectively. (**b**,**d**) The density of states of the monolayer β-AuSe using the PBE method and HSE06 method, respectively. (**e**–**g**) The 3D band structure for the monolayer β-AuSe in different views.

**Figure 3:**
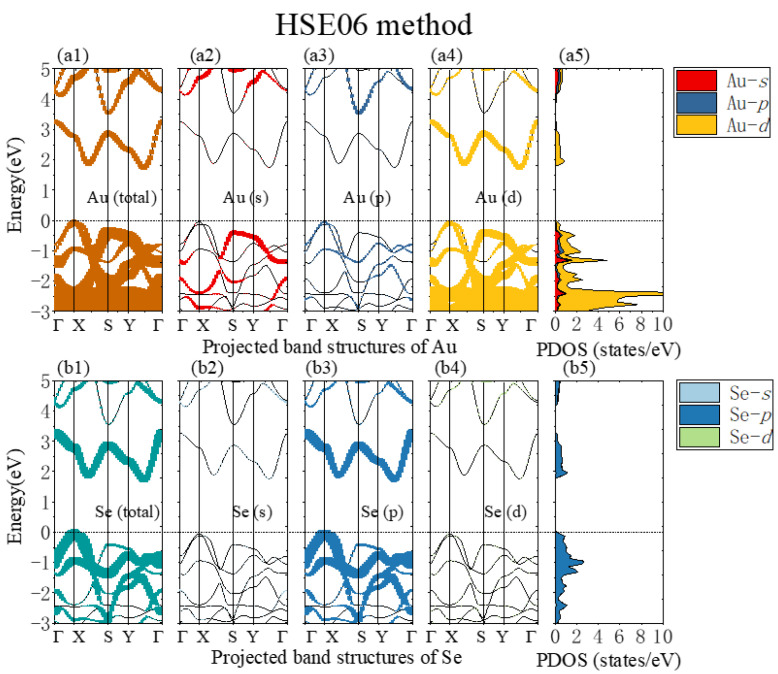
(**a1**,**b1**) The monolayer β-AuSe’s projected band structure of Au and Se using the HSE06 method. (**a2**–**a4**) The projected band structure of Au-*s*, Au-*p*, and Au-*d* electrons, respectively. (**b2**–**b4**) The projected band structure of Se-*s*, Se-*p*, and Se-*d* electrons, respectively. (**a5**,**b5**) The partial density of states of monolayer β-AuSe using the HSE06 method.

To start with, we studied the band structure and electron density of states (DOS) of strain-free monolayer β-AuSe (Figure 2). The BZ and high-symmetry k-points are shown in Figure 1g. Based on the calculated band structure and 3D band structure, we found that monolayer β-AuSe is an indirect band gap semiconductor (Figure 2e,f). The band gap was 1.21 eV and 1.8 eV using the PBE method and HSE06 method, respectively, which agrees with previous calculations [26,31,32,33]. Moreover, there was a significant band gap between CBM and the other conduction bands. Furthermore, the projected band structure and the partial density of states (Figure 3 and Figure S3) reveal that the CBM of monolayer β-AuSe is mainly composed of Au-*d* and Se-*p* electrons, and the VBM is composed of hybrid Au-*d*, Se-*p*, and bits of Au-*s* electrons.

**Figure 4:**
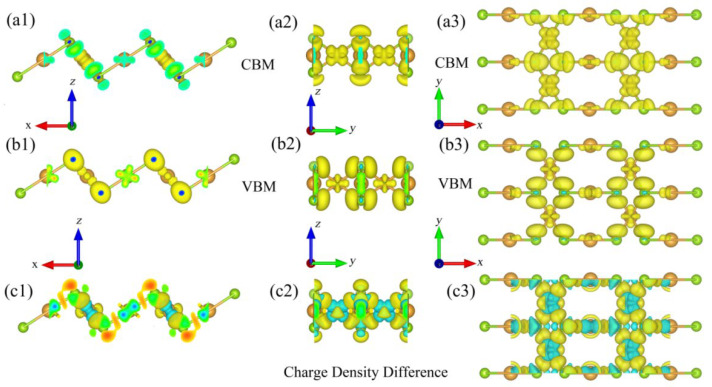
(**a1**–**a3**) Charge density (CD) of monolayer β-AuSe’s CBM in different views, where the isosurface level is set to 0.01. (**b1**–**b3**) Charge density (CD) of monolayer β-AuSe’s VBM in different views, where the isosurface level is set to 0.01. (**c1**–**c3**) The charge density difference of monolayer β-AuSe, where the isosurface value is set to 0.006. The blue and yellow in panels (**c1**–**c3**) represent losing and gaining electrons.

Next, to further study the interaction and bond nature between each atoms in monolayer β-AuSe, we calculated the charge density (CD) of CBM (Figure 4(a1–a3)) and VBM (Figure 4(b1–b3)) (the isosurface value is 0.01). We can see that the hole states of VBM were mainly provided by Au^(2)^, and a small part was provided by Au^(4)^. In comparison, the electronic states of CBM were mainly provided by Au^(4)^ and somewhat by Au^(2)^. In other words, the charge density of VBM was concentrated near Au^(2)^, while the charge density of CBM was located around Au^(4)^. 

Figure 4(c1–c3) shows the charge density difference of monolayer β-AuSe, where the isosurface value was set to 0.006, which can be defined by Equation (6). $\rho_{AuSe}$*,* $\rho_{Au}$, and $\rho_{Se}$ denote the charge density of monolayer β-AuSe, Au, and Se, respectively.

(6) $$\Delta \rho =\rho_{AuSe}-\rho_{Au}-\rho_{Se}$$

As shown in Figure 4(c1–c3), in monolayer β-AuSe, both Au and Se atoms lost and gained charge. The position where charge was gained was closer to Se atoms. Thus, we assumed that the bonds of Au^(4)^–Se and Au^(2)^–Se were composed of partial ionic bonds and partial polarized covalent bonds, and there were lone-pair electrons (LPEs) around Se. According to the *p–d* hybridization model proposed by Tang et al., the *–-d* hybridization between the metal-*d* and chalcogens-*p* bands will delocalize the wavefunction of the band edge states. The *p–d* hybridization could reduce the effective mass and improve the carrier mobility [42]. Combining our results and our predecessors’ [26,42], we suggest that the excellent carrier transport properties of the strain-free monolayer β-AuSe were due to the extremely small deformation potential mentioned by Gong et al., the unique bond nature, the existence of LPEs, and the influence of the *p–d* coupling between metal Au-*d* and Se-*p* bands around the VBM and CBM.

#### 3.1.3. Optical Properties

Exploring the application of 2D β-AuSe in the new energy field and photoelectric field requires the study of its optical properties.

**Figure 5:**
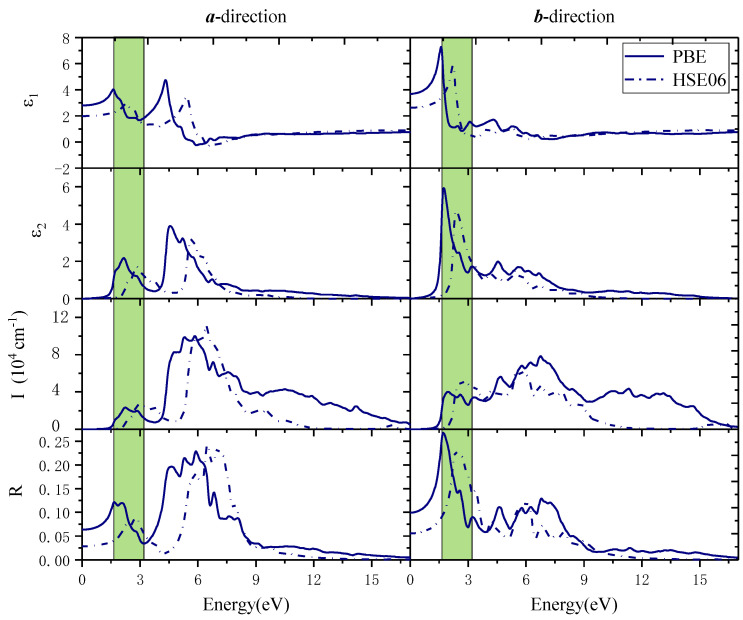
The calculated real part of the dielectric function (*ε*_1_), the imaginary part of the dielectric function (*ε*_2_) absorption coefficient (I), and reflection coefficient (R) in the ***a***-direction and ***b***-direction using the PBE method (solid line) and HSE06 method (dotted line). The area in the shaded area corresponds to the visible light region.

For strain-free monolayer β-AuSe, the complex dielectric function (*ε*), absorption coefficient (I), and reflection coefficient (R) were calculated (Figure 5). The shaded area in Figure 5 corresponds to the visible light region. In the ***a***-direction, when the PBE method is applied, the absorption edge was 1.49 eV. The first and second absorption peaks were located at 2.25 eV and 2.58 eV, and there was a shoulder peak at 1.74 eV. These peaks were all in the visible light region, and the absorption coefficient in the visible light region was up to 2.32 × 10^4^ cm^−^^1^. The maximum absorption peak was located in the UV region of 5.85 eV, and its absorption coefficient was 1 × 10^5^ cm^−^^1^. Its absorption coefficient decreased to zero when the energy exceeded 17 eV. Moreover, its static reflectance was 6%, and its reflection coefficient was less than 12% in the whole visible light region. Its maximum reflection peak appeared in the UV region of 5.9 eV, and the coefficient was 23%. In the ***b***-direction, the absorption edge was 1.27 eV. The first and second absorption peaks were at 1.97 eV and 2.58 eV. In the visible light region, the absorption coefficient reached 4.03 × 10^4^ cm^−^^1^. Its maximum absorption peak was in the UV region of 6.75 eV, and its coefficient was 7.83 × 10^4^ cm^−^^1^. The absorption coefficient dropped to zero when the energy was above 17 eV. Its static reflectance was 10%. Its maximum reflection peak was at 1.71 eV in the visible light region, and its maximum reflection coefficient reached 27%. In the visible light region, its reflection coefficient decreased gradually with the increase in photon energy, while in the UV region, its reflection coefficient was less than 13%. Furthermore, in the ***a***-direction, monolayer β-AuSe exhibited better transparency in the VIS region than in the UV region. Nevertheless, in the ***b***-direction, its transparency in the visible light was worse than that in the UV region. Therefore, monolayer β-AuSe has intense optical anisotropy.

When the HSE06 method was applied, the calculated optical properties were as shown by the dotted line in Figure 5. Along the ***a***-direction, its absorption edge was 2.05 eV. The first light absorption peak was at 2.93 eV, which was in the visible light region. Moreover, the peak value was 2.66 × 10^4^ cm^−^^1^, which was slightly higher than the results obtained by the PBE method. Its maximum absorption peak appeared in the deep UV region of 6.42 eV. Its static reflectance was 2.8%, and its reflection coefficient was less than 9% in the whole visible light region. Its maximum reflection peak was located in the UV region of 6.5 eV, and the maximum reflection coefficient was 24%. The absorption coefficient and reflection coefficient decreased to zero when the energy exceeded 12 eV. Along the ***b***-direction, its absorption edge was 1.71 eV. In the visible light region, its first absorption peak was at 2.68 eV with a peak value of 5.17 × 10^4^ cm^−^^1^, which was slightly higher than the results obtained by the PBE method. Its maximum absorption peak appeared at 5.9eV in the deep ultraviolet region. Its static reflectance was 5.6%. The maximum reflection peak was in the visible light region of 2.36 eV, and the reflection coefficient was less than 23%. However, the reflection coefficient was less than 15% in the UV region. When the energy exceeded 12 eV, the absorption coefficient and light reflection coefficient decreased to zero.

The results displayed a high similarity to the optical properties obtained by the PBE method and HSE06 method. The main differences between the results obtained by the two methods were the optical absorption edge, static dielectric constant, static reflectance coefficient, and the energy range of the principal peak values. These differences are mainly due to modifying the exchange and correlation energy by using the HSE06 hybrid functional method, which changes the band gap value.

We found that two factors cause the different optical characteristics in different directions: the low symmetry of the structure and the electrons in the ***a***-direction and ***b***-direction exhibiting different localization and charge densities. Thus, the anisotropic carrier concentration and charge density determined its difference in optical properties along the ***a***-direction and ***b***-direction.

Based on the above results, we conclude that monolayer β-AuSe is a promising candidate in the field of flexible polarization-sensitive optoelectronics, solar-blind photoelectric detection, environmental monitoring, high-efficiency directional optoelectronics, and even ultraviolet communication.

### 3.2. Strain-Controllable Properties of Monolayer β-AuSe

2D materials are favored by researchers in the optoelectronics, and new energy fields because of their distinct characteristics. Adjusting the photoelectric properties of 2D materials by strain is feasible and effective. Therefore, the study of the change of monolayer β-AuSe’s photoelectric properties tuned by strain is of great significance for its application in the future.

#### 3.2.1. Strain-Controllable Electronic Properties of Monolayer β-AuSe

**Figure 6:**
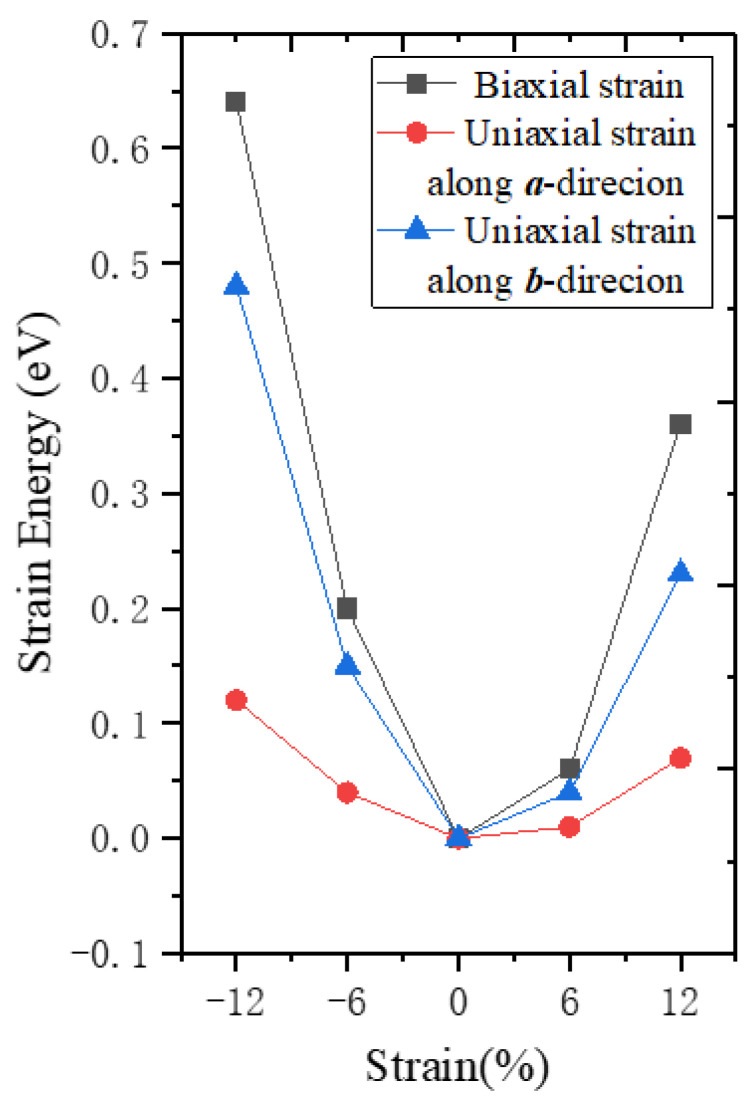
The strain energy under different strains.

First, we calculated the strain energy when different strains were applied (Figure 6). The strain energy is the difference between the strained and strain-free monolayer β-AuSe. Under a certain strain value, the calculated strain energy was lowest when the ***a***-directional uniaxial strain was applied, and highest when biaxial strain was exerted. For the same type of strain, the strain energy’s variation of tensile strain was smaller than that of the compressive strain. In other words, monolayer β-AuSe is most susceptible to deformation when it is subjected to ***a***-directional uniaxial tensile strain.

**Figure 7:**
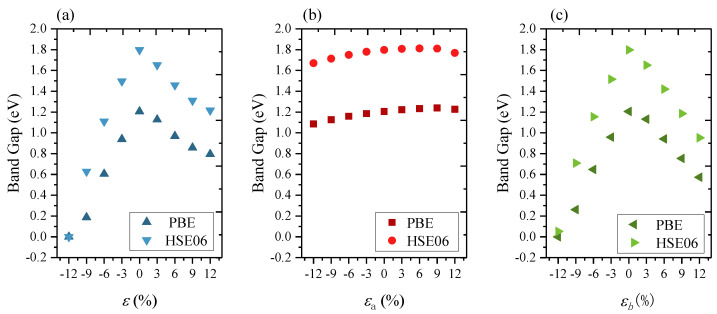
The changes in band gap when different strains were applied: (**a**) under the biaxial strain, (**b**) under the uniaxial strain along the ***a***-direction, and (**c**) under uniaxial strain along the ***b***-direction using the PBE and HSE06 methods.

For the strain-tunable electronic properties, the uniaxial and biaxial strain range from −12% compressive strain to 12% tensile strain was considered. The band gap variations of monolayer β-AuSe under different strains were calculated (Figure 7). The PBE and HSE06 methods were both adopted for our calculations. We found the same variation trend of the band structure and the band gap using these two methods.

**Figure 8:**
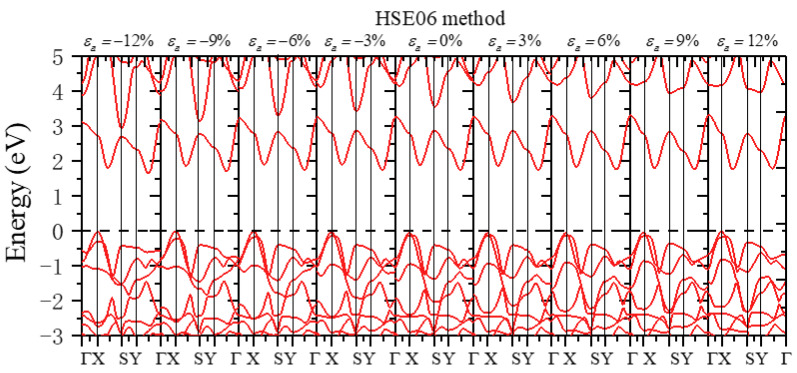
The band structure when different uniaxial strains along the ***a***-direction were applied using the HSE06 method.

We next discuss the change in monolayer β-AuSe’s electronic properties when applying the uniaxial strain in the ***a***-direction. First, the band gap of monolayer β-AuSe decreased when increased compressive uniaxial strain was applied along the ***a***-direction. The band gap increased when the uniaxial strain changed from *ε**_a_*** = 0 to *ε**_a_*** = 9%. Moreover, the band gap decreased when the strain increased from *ε**_a_*** = 9% to *ε**_a_*** = 12%. Secondly, the band gap decreased when uniaxial strain was applied along the ***b***-direction, regardless of the compressive or tensile strain. Moreover, the decreasing degree under compressive strain was more significant than that under tensile strain. According to the results obtained by the HSE06 method, the band gap of monolayer β-AuSe did not reach zero in the range from *ε**_b_*** = −12% to *ε**_b_*** = 12%. Thus, the monolayer β-AuSe could remain semiconducting under the uniaxial strain from *ε**_b_*** = −12% to *ε**_b_*** = 12%. Thirdly, the band gap of monolayer β-AuSe decreased when biaxial strain was applied. The band gap revealed a more noticeable decrease when compressive strain was applied. When the biaxial strain *ε* = −12% was applied, the band gap of monolayer β-AuSe became zero. Combined with the calculated band structure (Figure 8 and Figure S4), monolayer β-AuSe exhibited semimetallic properties under *ε* = −12%. Namely, a semiconductor–semimetal transition occurred when biaxial strain *ε* = −12% was applied.

**Figure 9:**
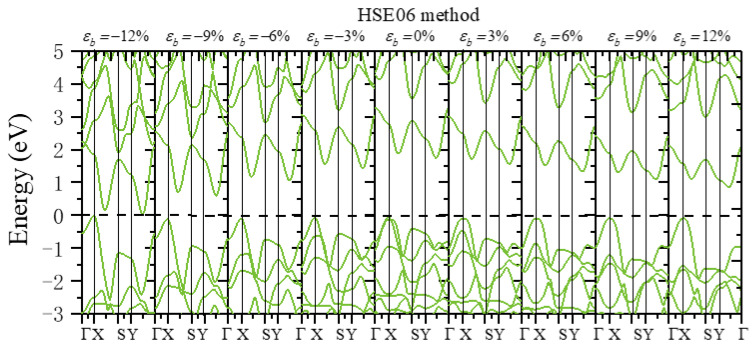
The band structure when different uniaxial strains along ***b***-direction were applied using the HSE06 method.

**Figure 10:**
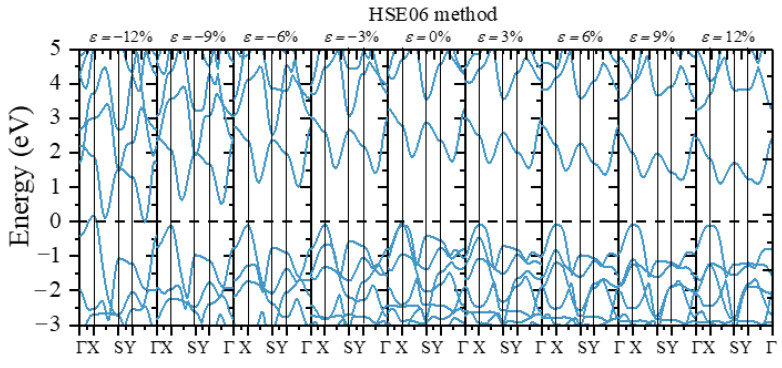
The band structure when different biaxial strains are applied using the HSE06 method.

More electronic properties of monolayer β-AuSe can be obtained from the calculated band structure (Figure 8, Figure 9 and Figure 10 and Figures S4–S6). First, when uniaxial strain in the ***a***-direction ranging from *ε**_a_*** = −12% to *ε**_a_*** = 9% was applied, the positions of VBM and CBM remained the same, and the value of the band gap changed. Moreover, the calculated strain-dependent change of band gap value was less than 0.16 eV using either the PBE or HSE06 method. When *ε**_a_*** = 12%, the CBM changed from the point between Y–Г to the point between X–Г. Thus, monolayer β-AuSe exhibited stable electronic properties under uniaxial strain along the ***a***-direction. In the conduction band, the most apparent variation appeared at the S point in the band, with energy higher than the band containing the CBM. According to the previous analysis, this position is mainly occupied by Au-*p* electrons. The energy of this position gradually increased, and this band was away from the band containing the CBM when applying the ***a***-direction uniaxial strain from *ε**_a_*** = −12% to *ε**_a_*** = 12% (Figure 8 and Figure S4). Second, when uniaxial strain along the ***b***-direction ranging from *ε**_b_*** = −12% to *ε**_b_*** = 12% was applied, the positions of VBM and CBM remained unchanged; only the value of the band gap changed (Figure 9 and Figure S5). As the strain changed from *ε**_b_*** = −12% to *ε**_b_*** = 12%, the bandwidths of CBM decreased. In the conduction band, the most apparent variation appeared in the band with energy slightly higher than the band containing the CBM. From *ε**_b_*** = –12% to *ε**_b_*** = 12%, this band gradually moved further away from the band of CBM. Thirdly, when biaxial strain, which ranged from *ε* = −12% to *ε* = 12%, was applied, the VBM and CBM were located at the same position. Only the value of the bandgap changed. In particular, when *ε* = −12%, the CBM overlapped with the VBM, indicating that a semiconductor–semimetal transition occurred (Figure 10 and Figure S6).

In general, monolayer β-AuSe’s electronic properties showed different variations when various strains were applied. The effect of uniaxial strain along the ***a***-direction had less impact on the electronic properties of monolayer β-AuSe compared with the uniaxial strain along the ***b***-direction and biaxial strain. A semiconductor–semimetal transition occurred when biaxial compression strain larger than *ε* = −12% was applied. However, all strains in the range of −12% to 12% had little effect on VBM and CBM. The main change in the VBM and CBM bands was the bandwidth. In addition, different strains all had a strong influence on the band, with slightly higher energy than the band of CBM in the conduction band. That is to say, all of the strains mainly impacted the Au-*p*, Au-*d*, and Se-*p* electrons, which were farther away from the forbidden region, but had little influence on the Au-*d* and Se-*p* electrons near the VBM and CBM. We assume that the partial ionic bonds, partial polarized covalent bonds, LPEs around Se of monolayer β-AuSe, and the *p–d* hybridization effect led to the changes in electronic properties due to strain but allowed for relatively stable electronic properties under different strains.

#### 3.2.2. Strain-Controllable Optical Properties of Monolayer β-AuSe

Next, we discuss the changes in the optical properties of monolayer β-AuSe when different strains are applied. In later sections, we discuss the calculated optical properties obtained by the PBE method since we focus on the variation trend of monolayer β-AuSe’s optical properties, and there is a high similarity between the optical properties of strain-free monolayer β-AuSe obtained by the PBE method and HSE06 method. Moreover, the calculation of the optical properties requires a large amount of computation time and resources, while the PBE method could save on computing time and resources. 

**Figure 11:**
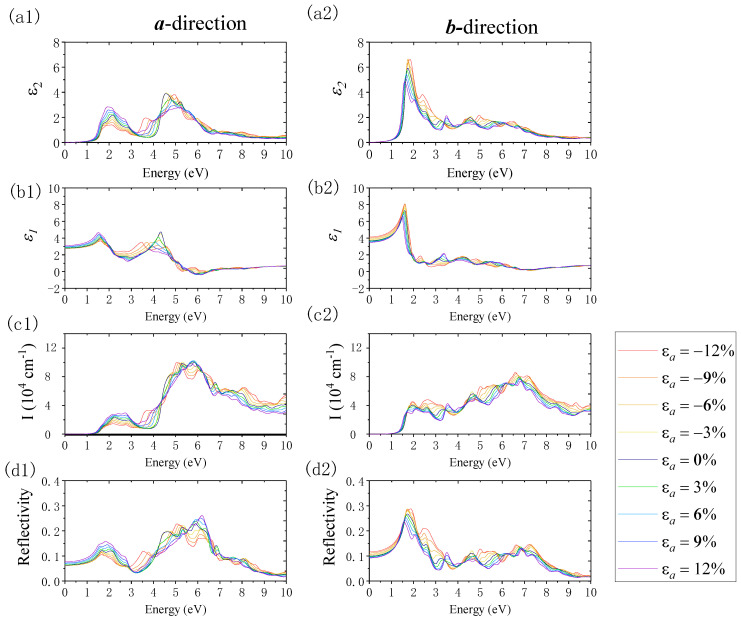
The variations in the imaginary part of the dielectric function (*ε*_2_), real part of the dielectric function (*ε*_1_), absorption coefficient (I), and reflection coefficient (R) when imposing the uniaxial strain along the ***a***-direction using the PBE method. Panels (**a1**–**d1**) and (**a2**–**d2**) show the results along the ***a***- and ***b***-directions, respectively.

Figure 11 shows the changes in optical properties when imposing the uniaxial strain along the ***a***-direction. Here, we focus on the absorption and reflection properties. In terms of absorption properties, in the ***a***-direction and ***b***-direction, the absorption edge revealed an inapparent redshift when exerting compressive strain. The absorption edge showed a more inconspicuous blueshift when applying tensile strain. The change in the absorption edge was negligible due to the inconspicuous change in the band gap when we applied the uniaxial strain along the ***a***-direction. When shifting the strain from *ε**_a_*** = −12% to *ε**_a_*** = 12%, in the ***a***-direction, the absorption coefficient in the visible light region increased, and the absorption range expanded. In the ultraviolet region, the maximum absorption peak revealed a blueshift, and its value remained unchanged. In the ***b***-direction, the absorption coefficient in the visible light region decreased, while in the ultraviolet region, the maximum absorption peak revealed a blueshift, and its value decreased. Furthermore, the absorption range in the ultraviolet region narrowed. In terms of the reflection properties, when changing the strain from *ε**_a_*** = −12% to *ε**_a_*** = 12% in the ***a***-direction, the static reflection coefficient and the reflection coefficient in the visible region increased, and the first reflection peak gradually shifted to the blue region. When the applied strain was compressive (from *ε**_a_*** = 0% to *ε**_a_*** = −12%), a new reflection peak arose in the 3–4 eV area, corresponding to the violet and near-ultraviolet region. With the increased compressive strain, this peak increased and shifted to red. In the deep ultraviolet region, the maximum reflection peak increased and shifted to red when the uniaxial strain changed from *ε**_a_*** = 0% to *ε**_a_*** = −12%, whereas it revealed a blueshift when the tensile strain changed from *ε**_a_*** = 0% to *ε**_a_*** = 12%. In the ***b***-direction, when applying strain from *ε**_a_*** = −12% to *ε**_a_*** = 12%, the static reflection coefficient and reflection coefficient decreased where the photon energy was located in the VIS region; meanwhile, the first reflection peak gradually shifted to red. The absorption edge showed a more inapparent blueshift when tensile strain was applied. In addition, when the subjected compressive strain increased from *ε**_a_*** = 0% to *ε**_a_*** = −12%, a new reflection peak appeared in the UV region in the range of 5–6 eV and 7–8 eV. This peak increased and shifted to red with the enhancement of the compressive strain.

**Figure 12:**
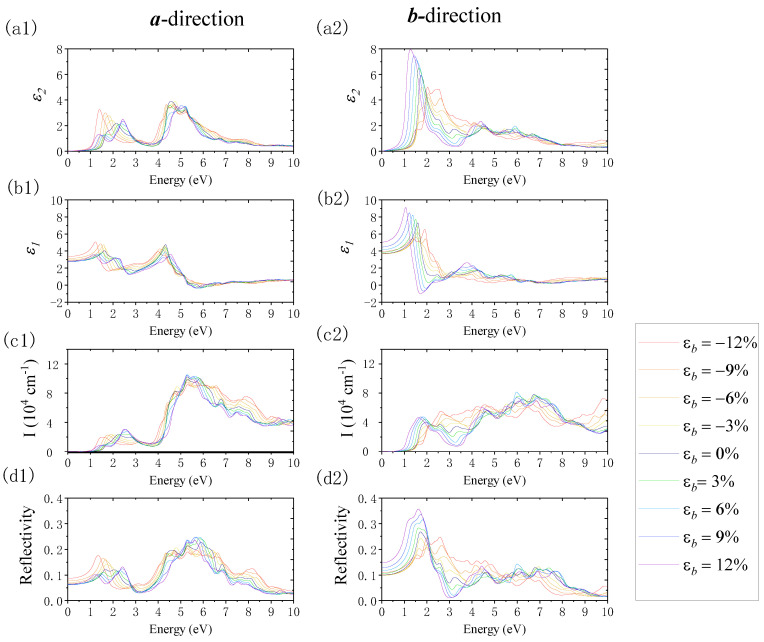
The changes in the imaginary part of the dielectric function (*ε*_2_), real part of the dielectric function (*ε*_1_), absorption coefficient (I), and reflection coefficient (R) when applying uniaxial strain along ***b***-direction using the PBE method. Panels (**a1**–**d1**) and (**a2**–**d2**) show the results along the ***a***- and ***b***-directions, respectively.

Figure 12 reveals the changes in monolayer β-AuSe’s optical properties under the effect of ***b***-directional uniaxial strain. In the ***a***-direction, when uniaxial compression strain from *ε**_b_*** = 0% to *ε**_b_*** = −12% was acting on it, the absorption edge and first absorption peak exhibited an obvious redshift. Meanwhile, the value of the first absorption peak decreased weakly. The absorption coefficient decreased in the visible region, where the energy was greater than the energy of the first absorption peak. Moreover, the width of the absorption in the visible region expanded. In the near-UV region, the maximum absorption peak’s value decreased with an increase in the compressive strain. The absorption coefficient increased with the enhancement of the compressive strain in the deep UV area of energy greater than 7 eV. In terms of the reflection properties, with the increase in compressive strain, the static reflection coefficient increased, and the first reflection peak increased and shifted to red. In the meantime, the reflection coefficient in the visible light region decreased, while the reflection’s energy range in the UV region expanded and the maximum reflection peak decreased. When uniaxial tensile strain from *ε**_b_*** = 0% to *ε**_b_*** = −12% was applied, its absorption edge exhibited an obvious redshift. Compared with the strain-free condition, with the increase in the tensile strain, the shoulder peak at 1.75 eV gradually changed to the first absorption peak and shifted to red. The value of the maximum absorption peak increased with a blueshift in the visible light region, while the peak value of the maximum absorption increased in the UV region. In terms of the reflection properties, with the increase in tensile strain, the static reflection coefficient decreased slightly, the first reflection peak decreased with a redshift, the second reflection peak increased with a blueshift, and the maximum reflection peak in the UV region increased. In the ***b***-direction, when a uniaxial compression strain of *ε**_b_*** = 0% to *ε**_b_*** = −12% was applied, the light absorption edge remained unchanged, the first absorption peak decreased, the second absorption peak increased, and the maximum absorption peak in the UV region decreased with a redshift. Moreover, the static reflection coefficient decreased slightly, the first reflection peak decreased and shifted to red, and the second reflection peak increased. The reflection coefficient increased in the near-UV region with energy less than 6.5 eV, whereas the reflection coefficient decreased in the deep UV region. The absorption edge shifted to red, and the first absorption peak increased when a uniaxial tensile strain of *ε**_b_*** = 0% to *ε**_b_*** = 12% was applied. In the same process, the second absorption peak decreased, and the maximum value of the absorption coefficient in the UV region increased. In addition, the static reflection coefficient increased, the first reflection peak increased and shifted to red, and the second reflection peak decreased.

**Figure 13:**
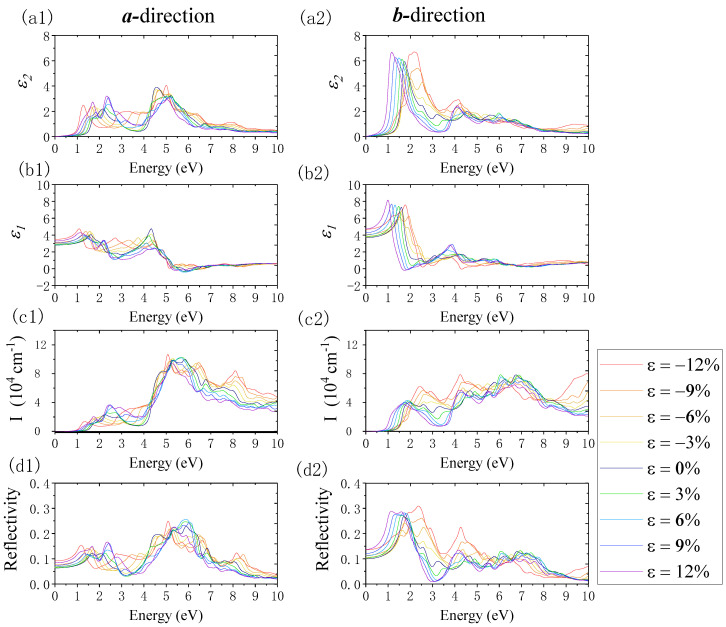
The changes in the imaginary part of the dielectric function (*ε*_2_), real part of the dielectric function (*ε*_1_), absorption coefficient (I), and reflection coefficient (R) when applying biaxial strain using the PBE method. Panels (**a1**–**d1**) and (**a2**–**d2**) show the results along the ***a****-* and ***b***-directions, respectively.

Finally, Figure 13 shows the change in optical properties under biaxial strain. In the ***a***-direction, when biaxial compression strain from *ε* = 0% to *ε* = −12% was applied, the absorption edge shifted to red, and the first absorption peak diminished and revealed a redshift. In the range of *ε* = 0% to *ε* = −6%, the second absorption peak decreased, whereas, within the range of *ε* = −6% to *ε* = −12%, the second absorption peak changed to a shoulder peak and the peak value increased. In the near-UV region, the absorption coefficient increased with the enhancement of the compression strain. The maximum absorption peak shifted to red in the deep UV region as the compression strain increased. The peak value increased with a biaxial strain change from *ε* = 0 to *ε* = −3%, decreased from *ε* = −3% to *ε* = −9%, and finally reached the maximum value when *ε* = −12%. In addition, the static reflection coefficient increased, and the first reflection peak increased and shifted to red with the increase in the compression strain. In the visible light region, the reflection coefficient increased in the red and blue–violet range and decreased in the green light region as the compressive strain increased. In the UV region, the maximum reflection peak exhibited a reducing, redshift trend when the biaxial strain changed from *ε* = 0% to *ε* = −9%. The maximum reflection peak increased and shifted to red when the strain was above *ε* = −9%. In the ***b***-direction, when the compression strain changed from 0% to −9%, the absorption edge was nearly unchanged, whereas, when *ε* = −12%, there was a redshift of the absorption edge. The first absorption peak revealed a decreasing trend, accompanied by a blueshift in the strain range of *ε* = 0% to *ε* = −6%. Moreover, the first absorption peak changed to a shoulder peak and exhibited a redshift trend in the strain range of *ε* = −6% to *ε* = −12%. Moreover, the second absorption peak increased with the increased compression biaxial strain. In the UV region, the value of the maximum absorption peak decreased and shifted to red when *ε* changed from 0% to −9%. Furthermore, when *ε* = −12%, the maximum absorption peak’s value reached the maximum. In addition, the static reflection coefficient increased when the biaxial compression strain changed from *ε* = 0% to *ε* = −12%. The first reflection peak decreased when the *ε* changed from 0% to −9%. When *ε* = −12%, the first reflection peak became a shoulder peak. The second reflection peak increased with the increase in compressive strain. When *ε* was greater than −6%, the second reflection peak replaced the first reflection peak. The reflection coefficient, located near the UV region with photon energy less than 5.56 eV, increased when the compression strain increased. However, in the deep UV region, where the photon energy was greater than 5.56 eV, the reflection coefficient decreased with the increase in compression strain. However, generally, in the ***b***-direction, the value of the maximum reflection coefficient under different biaxial strains is located in the visible light region and does not exceed 31%, and the reflection coefficient in the UV region does not exceed 22%. 

Next, we discuss the influence of the biaxial tensile strain on monolayer β-AuSe’s optical properties. In the ***a***-direction, when the tensile strain changed from 0% to 12%, the absorption edge shifted to red, the first absorption peak increased, and the shoulder peak before the first absorption peak increased and gradually became the new first absorption peak. In the visible region where the energy was greater than 2 eV, the absorption coefficient increased, and the absorption peak’s width narrowed. In the UV region, the maximum absorption peak exhibited a redshift with increased tensile strain, and the peak value increased when changing *ε* from 0% to 6% and decreased from 6% to *ε* = 9%. In the deep UV region with energy greater than 5.83 eV, the absorption coefficient decreased with an increase in tensile strain. In addition, the static reflection coefficient increased with increasing tensile strain. The first reflection peak diminished along with a redshift when *ε* changed from 0% to 6%. The first reflection peak rose when change *ε* from 6% to 9%. The second reflection peak increased with the increase in tensile strain. Moreover, the reflection coefficient in the visible region was less than 17%. The maximum reflection coefficient increased with *ε* changes from 0% to 6% in the UV region. Furthermore, it decreased when *ε* changed from 6% to 9%. The reflection coefficient in the UV region is not exceeding 26%. In the ***b***-direction, with an increase in the biaxial tensile strain, the absorption edge revealed a redshift, and the first absorption peak decreased. In the photon region with energy from 2 eV to 4 eV, a reduction in the absorption coefficient occurred with increased tensile strain, while the maximum absorption peak decreased in the deep UV region with increasing the tensile strain. In addition, when the tensile strain increased, the static reflection coefficient increased, the width of the maximum reflection peak in the visible region expanded, the maximum reflection peak showed a redshift, and the peak value increased. The reflection coefficient was less than 29% in the visible light area. Furthermore, the reflection coefficient in the UV region did not exceed 14%.

Overall, the optical properties of monolayer β-AuSe exhibited anisotropy in different directions. Moreover, applying different strains led to various changes in the optical properties. These changes were closely related to the variations in the electronic properties under different strains. Experimentally, the following techniques can be used to fabricate and induce strain in two-dimensional materials: bending a flexible substrate, elongating the substrate, piezoelectric stretching, exploiting the thermal expansion mismatch, controlled wrinkling, and a comparison between different techniques to induce biaxial strain or uniaxial strain. Strain engineering can be used to modify the electronic and optical properties of 2D materials in a controlled manner [43]. In the future, 2D β-AuSe may be a promising candidate for polarization-sensitive optoelectronics, solar-blind photoelectric detection, environmental monitoring, high-efficiency directional optoelectronics, and even ultraviolet communication.

## 4. Conclusions

In summary, we explored the strain-tuned structural, electronic, and optical properties of monolayer β-AuSe using first-principles calculations. Monolayer β-AuSe is a novel 2D semiconductor with dynamic stability, and can maintain good stability at room temperature. In terms of the electronic properties, its VBM and CBM are composed of hybridized Au-*d* and Se-*p* states. Moreover, it remains semiconducting under different strains over a wide range. The optical properties of strain-free monolayer β-AuSe are anisotropic, making it a promising candidate for polarization-sensitive optoelectronics and high-efficiency directional optoelectronics. Furthermore, the maximum absorption coefficient peak is in the UV region, which means β-AuSe can be used in the field of solar-blind ultraviolet photoelectric detection and even ultraviolet communication. Additionally, it can maintain relatively stable optical properties under a high strain rate, indicating that monolayer β-AuSe is a promising flexible 2D photoelectric material. Finally, we suggest that the excellent carrier transport properties of the strain-free monolayer β-AuSe and the stable electronic properties of the strained monolayer β-AuSe originate from the p–d hybridization effect. Hence, our study provides computational and theoretical support for the application of 2D β-AuSe in the field of high-performance flexible nano-optoelectronics.

## Supplementary Materials

The following supporting information can be downloaded at: https://www.mdpi.com/article/10.3390/nano12081272/s1, Figure S1: The phonon dispersion spectrums for the bulk β-AuSe. The coordinates of high-symmetry k-points in the Brillouin zone of the bulk β-AuSe are as follows: Г(0, 0, 0); L(−0.5, −0.5, 0.5); I(−0.717, −0.283, 0.5); I’(−0.283, 0.283, 0.5); Z(0, 0, 0.5); F’(−0.442, −0.442, 0.68); F(−0.559, −0.559, 0.33); Y(−0.5, −0.5, 0); X’(−0.695, −0.301, 0); N(−0.5, 0, 0); Figure S2: AIMD simulation at 300K of snapshots of monolayer β-AuSe at 0 and 5 ps; Figure S3: Panels (a1) and (b1) are the monolayer β-AuSe’s projected band structure of Au and Se by using the PBE method. Panels (a2–a4) represent the projected band structure of Au-*s*, Au-*p*, and Au-*d* electrons, respectively. Panels (b2–b4) represent the projected band structure of Se-*s*, Se-*p*, and Se-*d* electrons, respectively. Panels (a5) and (b5) are the partial density of states of monolayer β-AuSe using the PBE method; Figure S4: The band structure when different uniaxial strains along the a-direction are applied using the PBE method; Figure S5: The band structure when different uniaxial strains along the b-direction are applied using the PBE method; Figure S6: The band structure when different biaxial strains are applied using the PBE method.
