# Supplementary material for: Tuning the Electronic and Optical Properties of the Novel Monolayer Noble-Transition-Metal Dichalcogenides Semiconductor β-AuSe via Strain: A Computational Investigation
Source: Nanomaterials (Basel). 2022 Apr 8;12(8):1272. doi: 10.3390/nano12081272 (PMC9031954; doi:10.3390/nano12081272)
Supplement: Supplementary file 1 [file nanomaterials-12-01272-s001.zip › nanomaterials-1628676-supplementary.pdf]

nanomaterials

Supporting Information

Tuning the Electronic and Optical Properties of the Novel Monolayer Noble-Transition-Metal Dichalcogenides Semiconductor β-AuSe via Strain: A Computational Investigation

Qing-Yuan Chen [1,*], Bo-Run Zhao [1], Yi-Fen Zhao [1], Hai Yang [1], Kai Xiong [2] and Yao He [3,*]

1 School of Physical Science and Technology, Kunming University, Kunming 650214, China; borunzhao@163.com (B.-R.Z.); zyfen0402315@163.com (Y.-F.Z.); kmyangh@263.net (H.Y.)
2 Materials Genome Institute, School of Materials and Energy, Yunnan University, Kunming 650091, China; xiongkai@ynu.edu.cn
3 Department of Physics, Yunnan University, No. 2 Green Lake North Road, Wu Hua Qu, Kunming 650091, China
* Correspondence: qingyuanchen212@163.com (Q.-Y.C.); yhe@ynu.edu.cn (Y.H.)

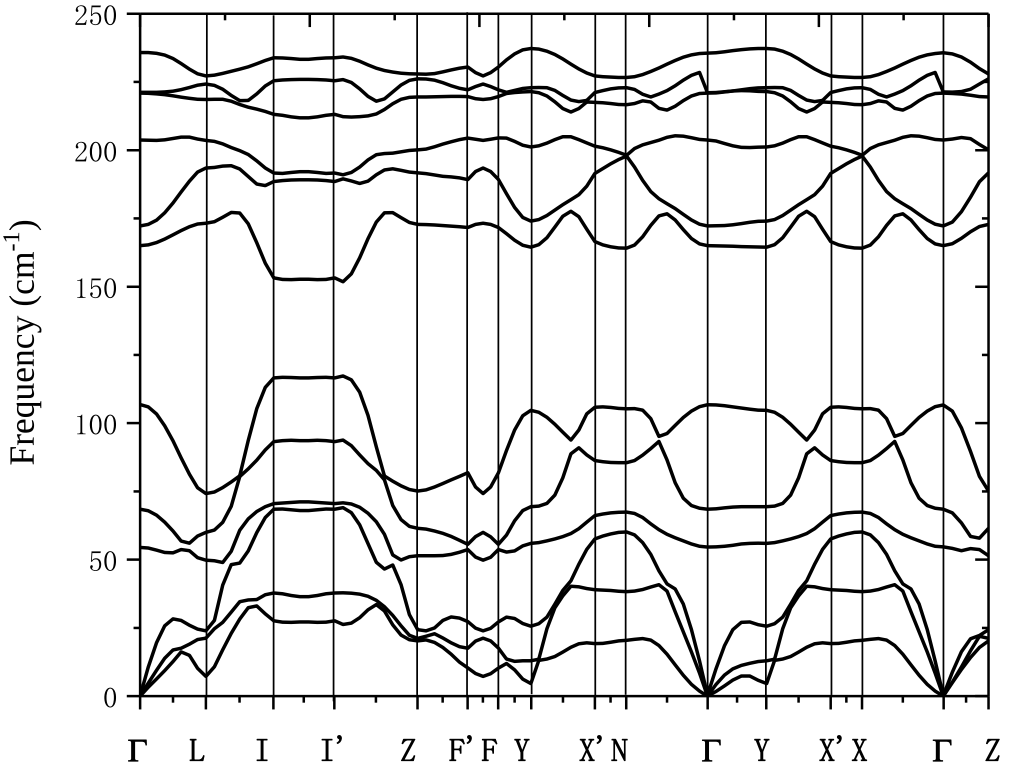

Figure S1. The phonon dispersion spectrums for the bulk β−AuSe. The coordinates of high−symmetry k−points in the Brillouin zone of the bulk β−AuSe are as follows: Γ(0, 0, 0); L(−0.5, −0.5, 0.5); I(−0.717, −0.283, 0.5); I'(−0.283, 0.283, 0.5); Z(0, 0, 0.5); F'(−0.442, −0.442, 0.68); F(−0.559, −0.559, 0.33); Y(−0.5, −0.5, 0); X'(−0.695, −0.301, 0); N(−0.5, 0, 0).

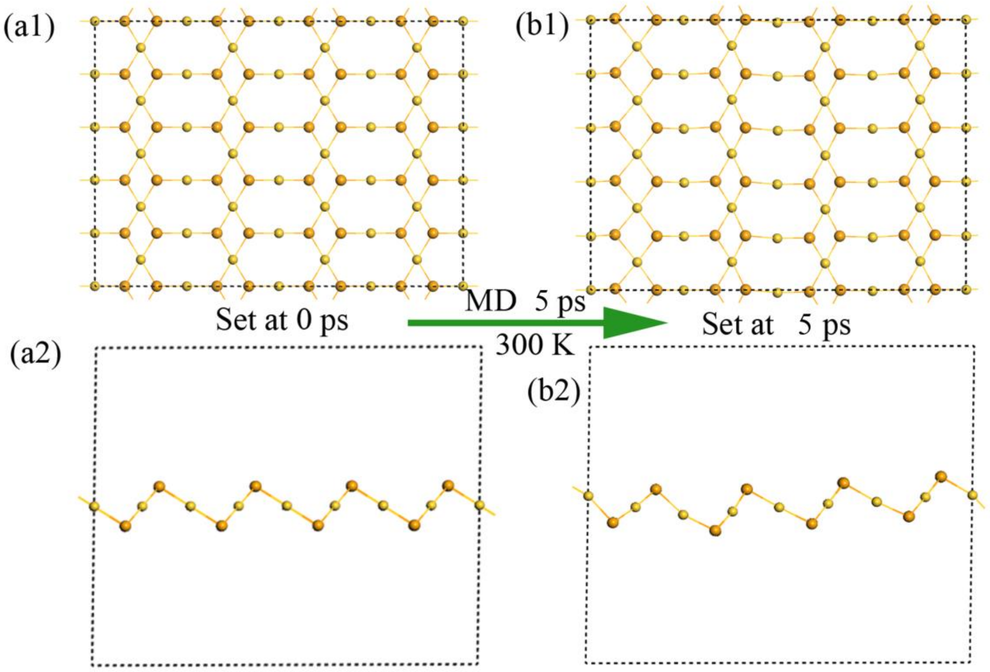

Figure S2. (**a1**,**a2**) is the top view and side view of the crystal structure of the monolayer β-AuSe at 0 ps. (**b1**,**b2**) is the top view and side view of the crystal structure of the monolayer β-AuSe when applying the AIMD simulation at 300K of snapshots of monolayer β-AuSe at 5 ps.

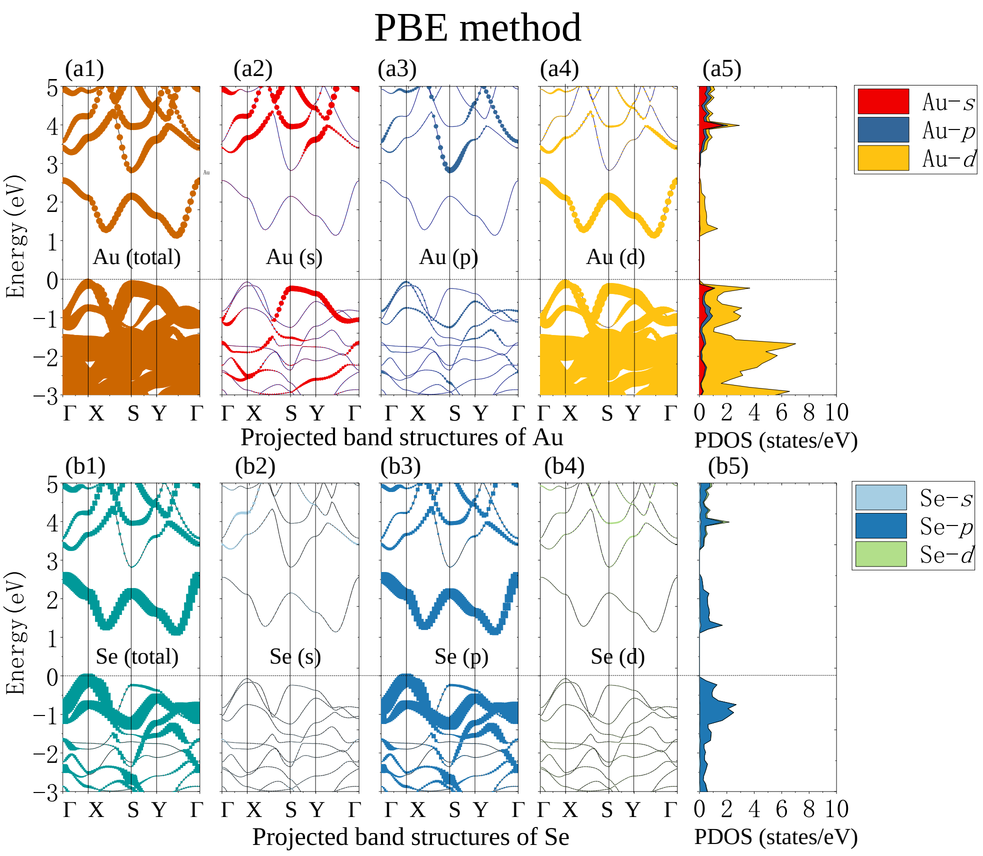

Figure S3. Panels (**a1**) and (**b1**) are the monolayer β-AuSe's projected band structure of Au and Se by using the PBE method. Panels (**a2**–**a4**) represent the projected band structure of Au-s, Au-p, and

Au-d electrons, respectively. Panels (**b2**–**b4**) represent the projected band structure of Se-s, Se-p, and Se-d electrons, respectively. Panels (**a5**) and (**b5**) are the partial density of states of monolayer β-AuSe by using the PBE method.

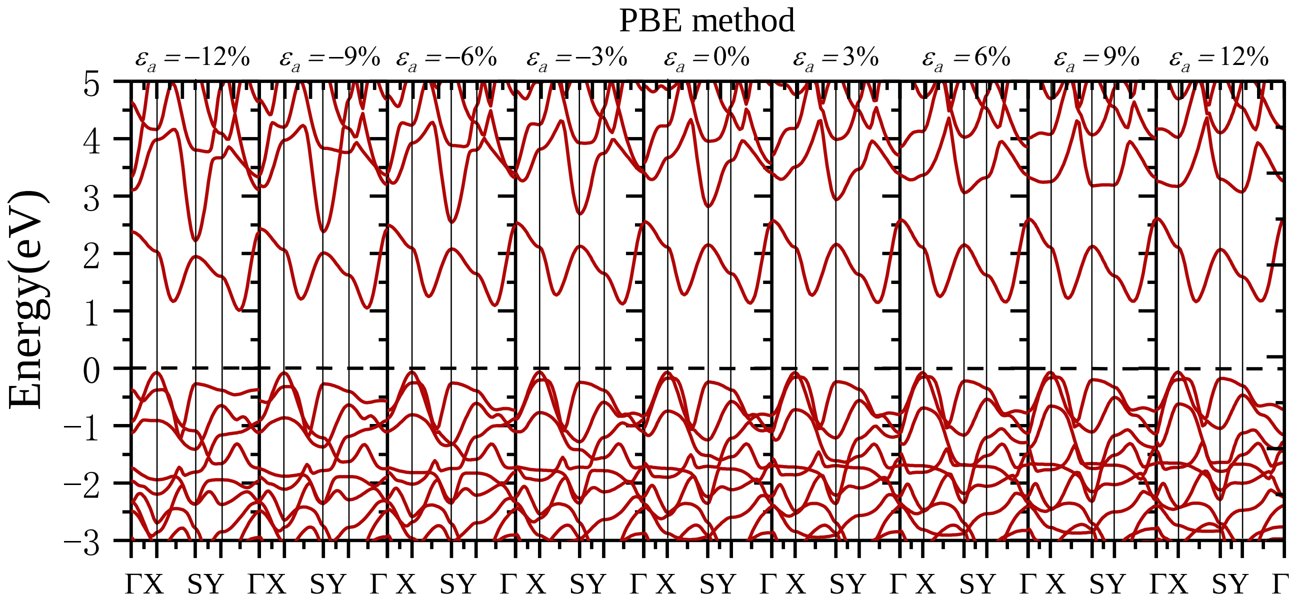

Figure S4. The band structure when different uniaxial strains along a-direction are applied by using the PBE method.

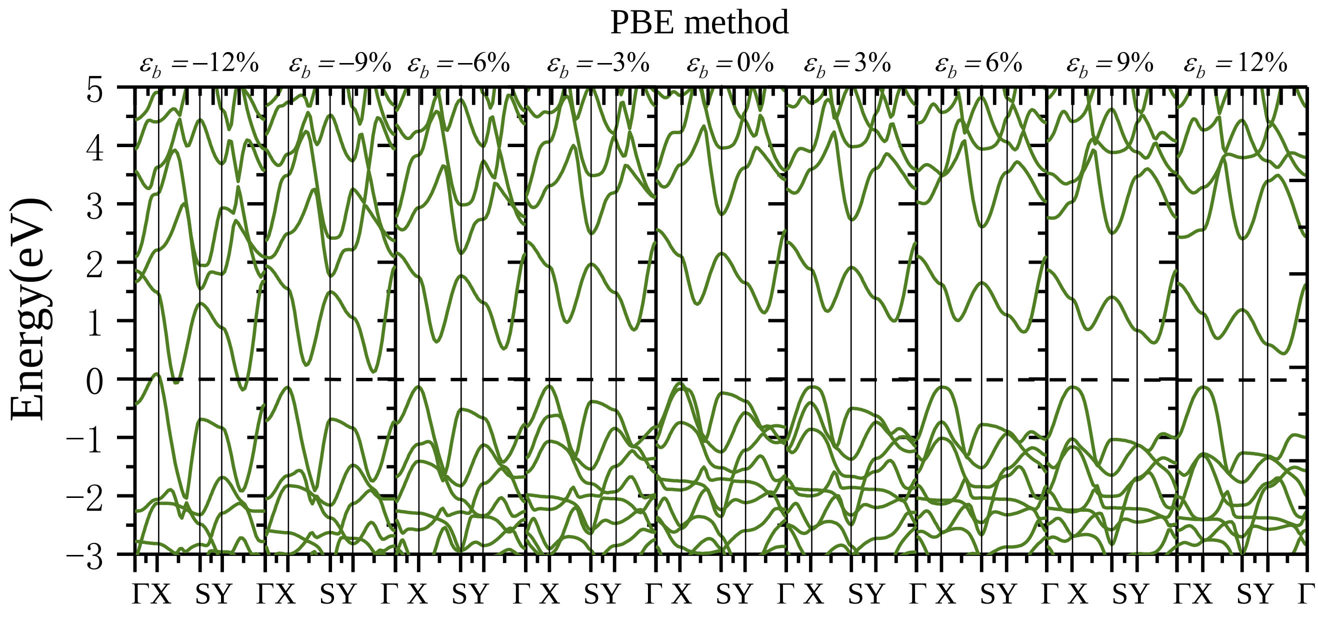

Figure S5. The band structure when different uniaxial strains along b-direction are applied by using the PBE method.

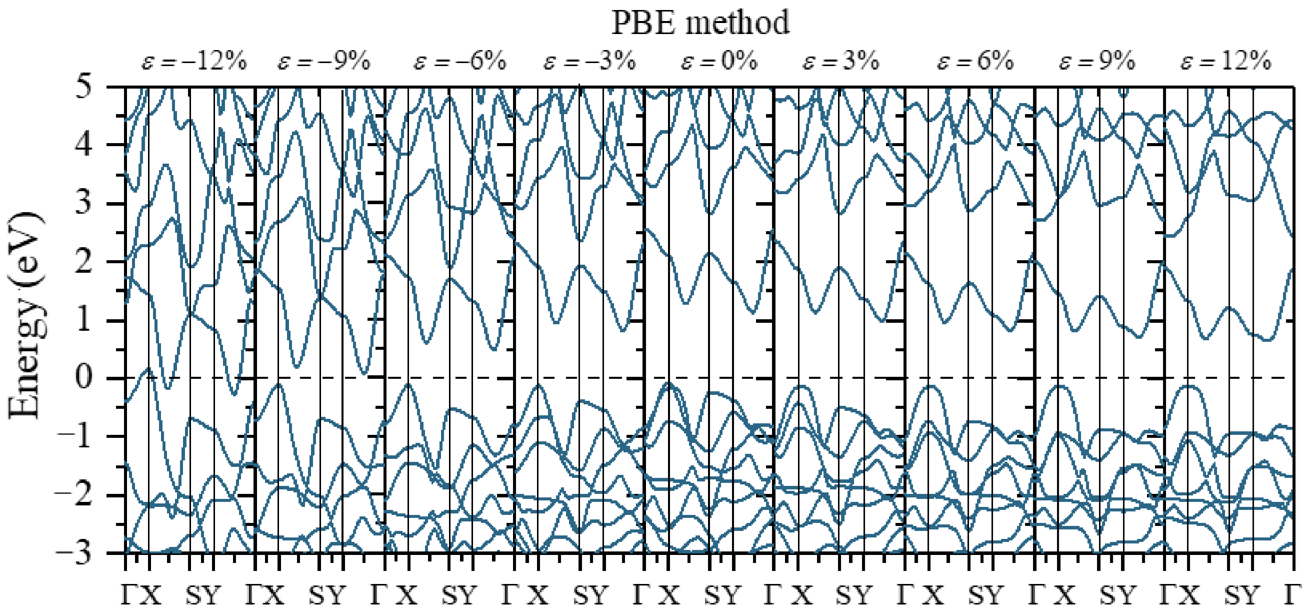

Figure S6. The band structure when different biaxial strains are applied by using the PBE method.
